# Occupational stressors and coping mechanisms among obstetrical nursing staff during the COVID-19 pandemic: a qualitative study

**DOI:** 10.1186/s12912-023-01557-6

**Published:** 2023-10-16

**Authors:** Julia Dobrowolski, Samia Chreim, Sanni Yaya, Serine Ramlawi, Alysha L. J. Dingwall-Harvey, Darine El-Chaâr

**Affiliations:** 1 Telfer School of Management, University of Ottawa, Ottawa, Canada; 2 Clinical Epidemiology Program, Ottawa Hospital Research Institute, Ottawa, Canada; 3 School of International Development and Global Studies, University of Ottawa, Ottawa, Canada; 4 The George Institute for Global Health, Imperial College London, London, UK; 5 Children’s Hospital of Eastern Ontario Research Institute, Ottawa, Canada; 6 Department of Obstetrics, Gynecology & Newborn Care, The Ottawa Hospital, Ottawa, Canada; 7 Department of Obstetrics and Gynecology, University of Ottawa, Ottawa, Canada; 8 School of Epidemiology and Public Health, University of Ottawa, Ottawa, Canada

**Keywords:** COVID-19, Health workforce, Nurses, Obstetrics, Occupational stress, Qualitative research

## Abstract

**Background:**

Due to heightened occupational stress throughout the COVID-19 pandemic, hospital nurses have experienced high rates of depression, anxiety, and burnout. Nurses in obstetrical departments faced unique challenges, such as the management of COVID-19 infection in pregnancy with limited evidence-based protocols and the unknown risks of the virus on pregnancy and fetal development. Despite evidence that obstetrical nurses have experienced high levels of job stress and a decrease in job satisfaction during the COVID-19 pandemic, there is less known about the working conditions resulting in these changes.

Using the Job Demands-Resources (JD-R) model, this study aims to offer insight into the COVID-19 working environment of obstetrical nurses and shed light on their COVID-19 working experiences.

**Methods:**

The study was conducted using a qualitative approach, with data collection occurring through semi-structured interviews from December 2021 to June 2022. A total of 20 obstetrical nurses recruited from the obstetrical departments of a tertiary hospital located in Ontario, Canada, participated in the study.

Interviews were audio-recorded, transcribed verbatim, and coded using NVivo. Data was analyzed using a theoretical thematic approach based on the JD-R model.

**Results:**

Four themes were identified: (1) Job stressors, (2) Consequences of working during COVID-19, (3) Personal resources, and (4) Constructive feedback surrounding job resources. The findings show that obstetrical nurses faced several unique job stressors during the COVID-19 pandemic but were often left feeling inadequately supported and undervalued by hospital upper management. However, participants offered several suggestions on how they believe support could have been improved and shared insight on resources they personally used to cope with job stress during the pandemic. A model was created to demonstrate the clear linkage between the four main themes.

**Conclusions:**

This qualitative study can help inform hospital management and public policy on how to better support and meet the needs of nurses working in obstetrical care during pandemics. Moreover, applying the JD-R model offers both a novel and comprehensive look at how the COVID-19 hospital work environment has influenced obstetrical nurses' well-being and performance.

## Introduction

The COVID-19 pandemic has had a profound impact on the birthing experiences of pregnant individuals and on the nursing staff who provide maternity care services. The pandemic drastically affected the roles, working environment, and overall well-being of the nursing staff working within the maternity care sector [1]. To reduce the spread of the virus, obstetrical departments needed to quickly adjust how they triaged, evaluated, and cared for patients. This included reducing in-person interactions between the birthing parent and care team, excluding family, friends, and support person(s) from both inpatient and outpatient care, and asking pregnant individuals to undergo universal COVID-19 testing [2].

Prior to the onset of the COVID-19 pandemic, nurses were already facing significant pressures and managing a wide range of work demands such as shift work, long work hours, and occasionally confronting demanding and emotionally taxing tasks [3, 4]. As a result, it was not uncommon for nurses to experience stress-related health problems including, but not limited to, burnout, musculoskeletal disorders, cognitive impairment, headaches, and trouble sleeping [5]. However, the unprecedented demands and unfavourable working conditions of the COVID-19 pandemic only exacerbated the mental and physical strain placed on nurses [6]. This was particularly the case for those working in obstetrical departments given the additional and unique challenges they faced, such as the management of COVID-19 infection throughout pregnancy with limited evidence-based protocols and the unknown risks of the virus on fetal development and vertical transmission [7, 8]. Furthermore, labour and delivery workers were considered a high-risk group for COVID-19 exposure due to the close proximity to aerosolization and blood products during the second stage of labour [9]. Notably, studies show that nursing personnel were at the greatest risk due to the considerable amount of time they spend providing one-on-one care to patients during the pandemic [10, 11].

A recent study exploring the psychological impact of COVID-19 on labour and delivery workers in Ontario, Canada, found that obstetrical nurses reported significant increases in job stress as well as a reduction in job satisfaction [12]. These findings align with another study which interviewed 14 perinatal nurse participants from the United States to determine how COVID-19 had impacted pregnancy and birth care. The perinatal nurses in the study reported they experienced significant psychological effects from the fear, stress, loss, and isolation caused by working during the pandemic [1]. Limitations of the studies investigating the COVID-19 experiences of labour and delivery workers include primarily quantitative research designs and a lack of focus on the experiences of obstetrical nurses working in Canada.

Aside from its impact on personal health, job stress among nurses has also been associated with decreased quality of care and patient satisfaction [13]. Furthermore, it can lead to increased rates of staff turnover, which results in greater costs incurred by the healthcare system [14]. Yet, a recent systematic review revealed a lack of evidence from studies, particularly qualitative, conducted during or after disease outbreaks that can help inform healthcare organizations about how they can better support and promote well-being among their staff [15]. There is, therefore, a strong need to better understand the circumstances resulting in job stress among obstetrical nurses during the COVID-19 pandemic and how they can be supported.

In this study we aim to offer insight into the COVID-19 working environment of obstetrical nurses in an Ontarian tertiary care centre and shed light on their COVID-19 working experiences. Specifically, we use the Job Demands-Resources (JD-R) model, developed by Demerouti et al. [16], as a framework to uniquely categorize obstetrical nurses' key stressors, coping strategies, and perceptions regarding hospital support. By doing so, it enables us to better understand the connection between obstetrical nurses' working environment and how it may affect their physical and mental well-being as well as job satisfaction. Moreover, it enables us to put in place recommendations for hospital upper management on how they can better support and meet the needs of obstetrical nurses during disease outbreaks moving forward. The study seeks to answer the following questions: (1) *What are the specific occupational stressors obstetrical nurses faced while working throughout COVID-19 and how did they cope with this stress?* (2) *From the nurses’ perspective, what additional support mechanisms could the hospital have offered to obstetrical nurses throughout the COVID-19 pandemic?*

## Background

The JD-R model is well established and commonly used in occupational health studies to investigate factors related to employees' work performance and general well-being [5, 17]. In light of the COVID-19 pandemic, it has also been proven to be helpful in categorizing novel working conditions as job demands and therefore used to identify factors contributing to job stress, such as COVID-19 transmission [18]. However, to the best of our knowledge, there have been no studies that have applied the JD-R model to better understand the COVID-19 occupational circumstances of labour and delivery workers in Canada.

The JD-R model was first published by Demerouti et al. [16] in an effort to comprehend the factors leading to burnout; it focused primarily on the following two major components: job demands and job resources. Job demands, which can become job stressors when extreme, refer to aspects of the job that require sustained physical/mental effort and are associated with certain physiological and psychological costs [16]. Job resources refer to resources provided by the employer that can reduce job demands and their associated negative physiological and psychological costs [16]. Over the years, various scholars continued working on the JD-R model, however, one of the most important extensions was the inclusion of personal resources by Xanthopoulou, Bakker, Demerouti and Schaufeli [19]. In a study of 714 Dutch employees, Xanthopoulou et al. [19] found that personal resources mediated the relationship between job resources and exhaustion and, thus, the JD-R model was expanded. In our study we chose to rely on the updated version as we believe it will offer a more comprehensive look at factors potentially impacting the well-being of obstetrical nurses during COVID-19.

The primary premise of the JD-R model is that extreme job demands exhaust employees' mental and physical needs, and if mixed with a lack of job resources, can lead to a state of exhaustion and eventually result in health problems. Moreover, the interaction between job demands and resources can indirectly impact organizational outcomes. A secondary premise of the JD-R model is the effect that job demands and job resources have on strain and motivation. It is assumed that job resources have motivational potential and lead to high work engagement and commitment, whereas overwhelming job demands can strain individuals, resulting in job-related anxieties, health complaints, and decreased job performance. Understanding the dynamics between job demands and job resources can help highlight the COVID-19 work circumstances resulting in a negative physical and mental strain among obstetrical nurses.

It should be noted that greater organizational support, particularly during times of increased job stress such as the COVID-19 pandemic, can serve as a protective factor against decreased physical and mental well-being. To ensure adequate job resources are offered to obstetrical nurses during major disease outbreaks, hospitals could use stress management interventions, which are defined as programs or activities initiated by an organization that focus "on reducing the presence of work-related stressors or on assisting individuals to minimize the negative outcomes of exposure to these stressors" (Ivancevich et al., [20], p.252). Organizational stress management interventions commonly used within the healthcare sector include reduced work hours, increased participation in decision-making, peer support groups, leadership development, team building, and improved communication [21]. Among nurses, organizational support has been found to help promote a positive work attitude, make ethical decisions, increase their commitment, and reduce turnover rates [22].

## Methods

### Study design

The study was conducted using a qualitative approach, with data collection occurring through in-depth semi-structured interviews. This approach allowed for direct one-on-one engagement with individual participants, and when paired with various probing techniques, helped offer a deeper understanding into the unique COVID-19 experiences and perspectives of labour and delivery nurses.

### Setting and participants

Purposive sampling was used to recruit participants from obstetrical departments housed in a tertiary hospital located in Ontario, Canada. Inclusion criteria required participants to have worked for a minimum of six months prior to the COVID-19 pandemic and a minimum of two months during COVID-19 (start date of the pandemic being March 11^th^, 2020). This ensured that participants involved would have knowledge about the working environment prior to COVID-19 and thus would be able to address specific changes that arose due to the pandemic. A total of 20 nurses were recruited from obstetrical units across two hospital campuses. Code saturation [23] was used to ensure a sufficient sample size was achieved, which occurred at interview number 16.

### Recruitment

To notify perinatal nurses of the ongoing study, department managers were asked to share recruitment information with their staff via email. Information shared by managers included study overview, eligibility criteria, potential risks, and details on how to privately contact the research team if interested in participating. Moreover, members of the research team would also visit the obstetrical departments to hand out study information sheets which helped boost recruitment. Participation was confidential to the research team and nursing managers were not aware of an individual's involvement in the study.

### Data collection

Given the nature of the ongoing COVID-19 pandemic at the time of the study, data collection occurred virtually. Semi-structured interviews of approximately 40-min were conducted between December 2021 to June 2022 over a video-conferencing platform. An interview guide was used to ensure the same questions and relevant topics were pursued with each participant [24]. Interview questions were open-ended and non-leading, reducing the possibility of introducing interviewer bias and allowing participants to express the opinions they believed to be most important [24]. Participants were asked about their experiences and perceived stressors of working throughout COVID-19, the coping strategies they used to mitigate the additional job stress experienced during the pandemic, and their perceptions of the resources provided by the hospital. All interviews were conducted by the first author, a female graduate student with research experience in qualitative methods. No other people were present during the interview. The interviews were recorded, transcribed verbatim, and then anonymized.

### Data analysis

Theoretical thematic analysis was used to identify, organize, describe, and report themes from the data [25]. The initial coding framework was based on the elements of the JD-R model and any data not reflected in the model was coded using an inductive approach. Specifically three of the top-level codes, Job Stressors, Personal Resources, and Constructive Feedback Surrounding Job Resources, were pre-determined to reflect components of the JD-R model. The remaining top-level code, Consequences of Working During COVID-19, and all subcodes were formed inductively from the data. Initial codes, which consisted primarily of descriptive and in-vivo codes, were applied to help condense the data into analyzable units. For example, the in-vivo code "working short" was used as it captured the concept of working with insufficient staff and was the terminology most often used by participants. Once the initial coding process was complete, merging and sorting the various codes was undertaken to condense units into more abstract themes. For example, "helping with COVID-19 research", "enforcing COVID-19 policies", and "increased patient support" all contributed to the nurses' increasing workload, and therefore were grouped together under the theme of "increased workload". Themes and subthemes were then reviewed using an iterative approach moving between the transcripts and various coding levels to ensure they were supported by enough data and not overly diverse [26]. To aid in data management and quote retrieval, NVivo qualitative data analysis software, released in March 2020, was used throughout analysis [27]. Table 1 shows the relationship between the initial codes, subcodes, and the main themes discovered.

**Table 1:** Main themes, subcodes, and initial codes of the study

Initial Codes	Subthemes	Main Themes
Enforcing policies	Increased workload	Job Stressors
Helping with COVID-19 research		
Increased patient support		
Increased acuity (COVID-19 patients)		
Supporting junior nurses		
Equipment/supply shortages		
"Working short"		
"Bringing it home to family"	Fear of COVID-19 transmission	
Transmission between colleagues		
"Workplace self-isolation"		
Patients lying about symptoms		
Reduced quality of patient care	Providing proper patient care	
PPE^a^ (delays care and negative interaction)		
Equipment shortages		
Physical discomforts (PPE^a^)	Overwhelming physical demands	
Missing breaks		
No vacation		
"Burnt out"	Burnout	Consequences of Working During COVID-19
Exhaustion		
Loss of job satisfaction		
Drop in unit morale		
"Just a number"	Feeling undervalued	
Lack of recognition/appreciation		
"Labour and Delivery always forgotten"		
Vaccine rollout issue		
"In the trenches"	Feeling inadequately supported	
Lack of support		
Nursing week		
No more picking up shifts	Career modification	
Reduce work hours		
Changing career		
Lack of nurses	Concern for future of healthcare	
Future of healthcare		
Gardening	Personal hobbies	Personal Resources
Exercise		
Knitting		
Reading		
Sports		
Family time	Support from family and friends	
Pets		
Talking with friends		
Relying on my colleagues	Colleague support	
"Venting"		
Positivity on shift		
Use of humour		
Love my job	Love for the job	
Love labour and delivery		
Mental health days	Improving mental and physical support	Constructive Feedback Surrounding Job Resources
On site counsellors		
Increase debriefing sessions		
Huddles	Improving communication	
Main system to keep everyone updated		
Updates through electronic medical record system		
Workshops	Improving retention	
Team building		
Increase recognition/appreciation		
More breaks/vacations		
More flexibility in scheduling		
Free parking	Positive feedback	
Reduced visitors		
Screening		
"Beyond the hospitals control"		

^a^Personal Protective Equipment

### Trustworthiness

We followed several steps to ensure the quality of our study [28]. Three of the authors worked closely together during the analysis process to identify codes and themes, meeting to discuss and review the data regularly. If the consensus of themes was not achieved, differences in points of view were resolved by returning to the data. Once all differences were resolved and the code list stabilized, the final codes and themes were applied to all interviews. We also sought member checks to ensure that the findings were deemed credible by participants [29]. We sent copies of the Results section to all participants, which allowed them to review the data and provide feedback [28]. One participant responded indicating that supply chain issues had become a major issue and clarified that it should be included under the Job Stressors theme. Thus, the Results section was updated to include their feedback. Finally, to help the reader follow our analysis, we provide rich descriptions of themes and incorporate extensive quotations in the Results section of this paper.

### Ethical considerations

This study was approved by the Ottawa Health Science Network Research Ethics Board (20200640-01H) as well as the University of Ottawa Research Ethics Board (H-12–21-7533). Each participant received a study information sheet which disclosed the study's purpose, risks, and benefits. Given the nature of questions asked during the interview, risks included feeling uncomfortable or overwhelmed when reflecting on stressful workplace experiences. The study information sheet listed three resources, such as support pages for COVID-19 frontline staff, that participants could have utilized if needed. Moreover, it was made clear that participation was voluntary and that participants had the option to withdraw or stop the interview at any point. Using a verbal consent script, participants provided informed consent prior to starting the interview. To protect participant identities, interview transcripts were anonymized. Each participant was compensated with a $20 gift card, delivered electronically to a local coffee shop as an appreciation token for their time.

## Results

**Fig. 1:**
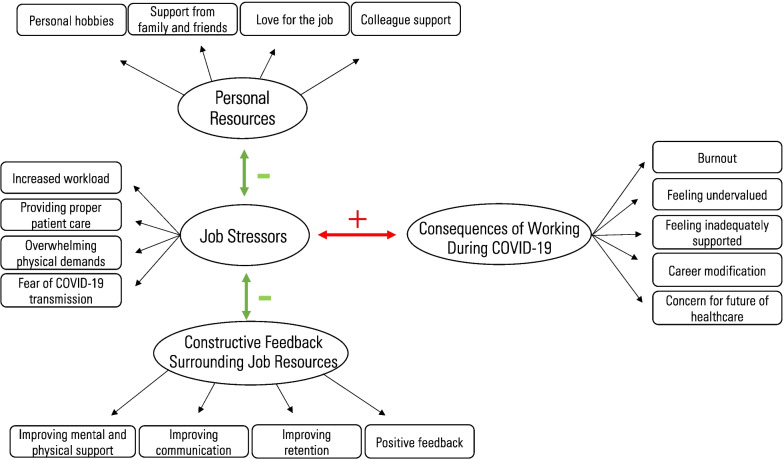
Model showcasing how personal resources and the COVID-19 hospital work environment has influenced obstetrical nurses' well-being and performance

A total of 20 obstetrical nurses were interviewed in this study. The nurses' years of experience working in the field of labour and delivery ranged from 3 to 23 years. The average interview length was approximately 40 min, with the shortest interview lasting 20 min and the longest lasting 64 min. Theoretical and inductive thematic analysis revealed the following 4 main themes: (1) Job Stressors, (2) Consequences of Working During COVID-19, (3) Personal Resources, and (4) Constructive Feedback Surrounding Job Resources. To better understand how the COVID-19 hospital work environment has influenced obstetrical nurses' well-being and performance, a model (Fig. 1) was created displaying the interaction between the four main themes.

### Theme 1—Job stressors

This theme captures the overwhelming job demands (i.e., stressors) that arose as a direct result of the COVID-19 pandemic. These include increased workload, fear of COVID-19 transmission, providing proper patient care, and overwhelming physical demands.

#### Increased workload

Participants expressed they felt overwhelmed trying to manage the various additional tasks brought on by the COVID-19 pandemic. For example, one of the most time-consuming tasks reported by the participants was having to constantly enforce and explain COVID-19 policies to anyone wishing to enter the labour and delivery units. However, participants noted they struggled to enforce certain policies more than others, such as having to tell a mother she will have to deliver alone or that she cannot see her baby if awaiting for COVID-19 test results: "I had people whose babies were dying or their babies died and they couldn't go see them in NICU because they were waiting for their COVID tests to come back" (P4). Additionally, sending home a birthing parent's support person only further increased nurses’ workload as the birthing parent now relied on them as their sole support: "When they weren't allowed visitors, your role really increased because you're no longer just the nurse, you're also their primary support person" (P16). Other reported tasks that arose during the pandemic were assisting with COVID-19 research, trying to stay up to date on all the changing protocols and procedures, cleaning rooms after COVID-19 patients, and finding workarounds due to the supply chain issues that caused medication and equipment shortages (i.e., epidural kits, amino hooks, saline bottles, monitoring belts). The strain of having an increased workload was further exacerbated given that obstetrical nurses frequently had to work short staffed due to the unique skillset required to work in the field of labour and delivery: "In medicine if 10 nurses were on isolation, they could pull from surgery, they could pull from other places, but nobody can do our job because we're so specialized" (P13). This also meant that obstetrical nurses frequently had to take on a greater number of labouring individuals or mother and baby dyads per shift than normally allocated to them.

#### Fear of COVID-19 transmission

The fear of picking up the COVID-19 virus at work and transmitting it to family, friends, and colleagues was a major stressor for obstetrical nurses. This fear was exacerbated given the unique protocol for triaging pregnant individuals in the hospital.

In labour and delivery there is a triage unit, it's kind of like an emergency department so people come in from anywhere and then you have to immediately think - do they have COVID? So for a long time we assumed everyone had it, as soon as they would sniffle, we would be paranoid. (P11)

Participants also highlighted they worried about transmitting the virus to patients given they often had to care for those with decreased immunity: "It was really uncomfortable because my job is taking care of people at their most vulnerable like newborns with no immune systems or mothers during C-sections with an open abdomen" (P13). The fear of COVID-19 transmission was heightened when nurses noticed that expecting individuals and/or their support team would frequently lie or omit COVID-19 symptoms to have their desired birthing experience.

#### Providing proper patient care

Participants reported that working short while having an overwhelming workload meant they often felt they had to rush through care. Consequently, participants were concerned about the quality of care they could provide and even feared they could lose their nursing licence.

I just want to be able to stay safe, take care of patients and not lose my license or think that I could lose my license every shift that I'm on - I don't know if I'm going to do something or not do something that my patient requires because I can't be cloned. (P2)

Participants mentioned that equipment shortages also had a significant negative impact on care and that personal protective equipment hindered their ability to quickly reach labouring patients.

It [personal protective equipment] really slows down the pace of the day and sometimes labour and delivery there's no time - the baby's coming. I feel people were unsure of how to protect themselves - if I take the time to dress properly, that baby's going to come out without me - do I chance it and just go in? (P18)

#### Overwhelming physical demands

Participants often emphasized that physical demands at work were becoming unbearable during the COVID-19 pandemic. For example, working 12-h shifts wearing full protective gear was reported as being extremely challenging and uncomfortable.

Wearing all that [personal protective equipment] for a 12-hour shift is extremely challenging (...) It's almost like going to a gas chamber where you would have to work wearing all that and you're sweating underneath and you still have to perform your duties. (P6)

Moreover, the inability to take breaks due to staffing issues or capacity limitations of break rooms often left nurses feeling physically unwell on shift. Participants often stressed that it was extremely difficult to receive any sort of vacation time granted over the course of the pandemic which made it more difficult to cope with the overwhelming physical demands as they had no time for respite.

### Theme 2—Consequences of working during COVID-19

This theme covers the impact, both physical and emotional, on frontline obstetrical nurses caused by working during the COVID-19 pandemic. There are 4 main subthemes: burnout, feeling undervalued, feeling inadequately supported, career modifications, and concern for the future of healthcare.

#### Burnout

Participants reported feeling mentally, physically, and emotionally exhausted from the working conditions experienced in labour and delivery during the COVID-19 pandemic. They noted that they struggled to feel the same level of job satisfaction as they did pre-pandemic and even noticed they were feeling less empathetic towards patients: "We are so burnt out that we just don't care anymore—we still care for our patients, but we've just given so much that I feel now it's just less" (P11). Loss of sleep, increased anxiety, lack of motivation and the inability to maintain a healthy work-life balance were other symptoms of burnout reported by participants. Furthermore, there was a severe sense of dread about going to work expressed by participants: "We've really stretched ourselves thin, so when you come home you just sit on the couch, and you can't even move your limbs and all you're thinking is I just wanna die so I don't have to go back" (P6).

#### Feeling undervalued

Participants indicated that they felt severely underappreciated and undervalued while working in labour and delivery during the pandemic. The concept of feeling disposable was common amongst those interviewed for the study: "We feel like we're numbers. We're just another person filling the numbers to quotas. I don't feel like we're valued" (P2). Moreover, participants felt that because of their work in the field of obstetrics, they were often overlooked by the hospital as they believed the department was often neglected.

I feel like we're in an area that's always forgotten because we are [perceived as] *"the happy area, nothing bad happens. Everyone just comes in and gives birth and leaves."* I felt like there was just a lot of misinformation about what we actually do. I felt like they [hospital management] just didn't know what we dealt with. (P17)

Specifically, participants indicated that they felt neglected during the vaccination rollout by not being categorized as a high-risk group even though their department has both triage and operating room aspects, which increased the likelihood of virus exposure.

#### Feeling inadequately supported

Participants reported feeling inadequately supported by the hospital over the course of the COVID-19 pandemic. For example, participants highlighted they felt there was a strong sense of misunderstanding from upper management when it came to addressing the needs of frontline labour and delivery nurses.

It just feels like we are in the trenches and everyone else is so out of touch with what it really is like to be working in our unit and not attempting to help us, or if they are, they're throwing us little cookies or a pizza. We don't need a pizza. We need help. (P5)

They reported feeling particularly not supported during night and weekend shifts as they were often left without a response when seeking clarification about COVID-19 policies outside of regular management hours.

#### Career modifications

Participants disclosed that during the pandemic, they often sought to reduce the amount of time spent in the hospital either through reducing work hours or taking leave: "Now I'm rarely picking up overtime because I don't want to spend any more time in the hospital than I have to. I don't look forward to going to work" (P4). Moreover, it was not uncommon for participants to disclose that they were looking to change careers altogether as a result of the extreme stress and unfavourable working conditions brought on by the pandemic. Those who wished to change careers often stated they hoped to remain in healthcare, ideally within the field of maternal health, but emphasized that bedside labour and delivery nursing in a hospital had become too overwhelming and was no longer a sustainable career.

#### Concern for the future of healthcare

Given their experiences and firsthand knowledge of what was going on in healthcare during the COVID-19 pandemic, participants often reported they worried about the future of the Canadian healthcare system: "I'm concerned about the future of healthcare and who's going to work in the hospital" (P20). They noted they had never seen such a high staff turnaround in obstetrics and were particularly concerned about how the department can function without an adequate number of nurses. It was highlighted that the working conditions were unrealistic, and consequently, there was an inability to retain staff. Many participants specifically worried and feared for younger nurses, particularly those just starting their careers: "You see young nurses just starting their career and they're full of energy and enthusiasm and they crumble—it really makes you concerned that there is something wrong with the system" (P6).

### Theme 3—Personal resources

This theme refers to resources highlighted by participants that they personally used to help ease the stress and emotional burden of working in labour and delivery during the COVID-19 pandemic. Relying on the following 4 mechanisms were most common: personal hobbies, support from family and friends, colleague support, and love for the job.

#### Personal hobbies

Participants identified a large range of personal hobbies such as exercising, knitting, reading, gardening, listening to music, and engaging in team sports as outlets to help reduce the impact of stress. However, these activities were often noted as ways to help disconnect from work and improve one’s physical, mental, and emotional well-being: "I picked up exercising in January 2020, which has been super consistent till now. Physical activity—I definitely use that as an outlet to stay healthy and help with stress" (P17).

#### Support from family and friends

Relying on the support of friends and family was widely acknowledged amongst participants as an important aspect that helped reduce stress levels. Increasing family time, going on walks with friends, partaking in virtual game nights, and spending time with pets were mechanisms reported by participants which helped them decompress. Many participants highlighted the importance of having someone who is a good listener and a reliable helping presence: "My friends, my family are super supportive and a really good network that I can rely on to help with any stress I have" (P17).

#### Colleague support

Participants expressed that relying on their colleagues for support was an essential coping mechanism to manage the stress of working during the pandemic. The concept of "venting" to other nurses in the obstetrics department was key as those sharing similar experiences could easily empathize and understand each other’s emotional struggles.

I feel like the team has really pulled together and we just really lean on each other - you can vent to each other in a room where nothing leaves the room, no names are mentioned but you can sort of get your feelings out. Have a good cry together in a small personal space. (P7)

Participants mentioned they would try to avoid, when possible, talking about COVID-19 at work and instead focus on positive aspects of each other's personal lives. Furthermore, the use of humour was commonly mentioned as a means to improve the unit's morale.

#### Love for the job

One of the driving forces keeping certain participants motivated to continue coming to work and overcome various stressors with ease is the continuous love they have for the field of maternal care: "My job has changed drastically and it's something that I never anticipated feeling or being in a position of contemplating leaving the profession. Labour and delivery, birthing unit, women’s health—it's in my soul, it's who I am" (P5). Reminding themselves why they entered this profession and chose to work in this specific field helped many nurses cope: "I think I just kept trying to tell myself and remind myself how much you still enjoy this and keep going, that this too shall pass" (P18).

### Theme 4—Constructive feedback surrounding job resources

This theme focuses on suggestions directed to the hospital to improve job resources, which participants in turn believe could have improved the working conditions for obstetrical nurses working during the COVID-19 pandemic. The suggestions revolved around the following subthemes: improving mental and physical support, improving communication, improving retention, and positive feedback.

#### Improving mental and physical support

As perinatal nurses felt inadequately supported throughout the COVID-19 pandemic, participants identified several areas and resources that they believed could have been improved. Suggestions to improve wellness resources included offering mental health days, access to onsite stress management specialists and a greater number of debriefing sessions on the unit. Another key resource would have been receiving more physical help on the unit. Acknowledging it was difficult to hire/retain obstetrical nurses, participants said simply having people who could have helped with non-nursing tasks would have been a big relief. For example, having those who work in management come down and help relieve strain on the unit.

Things that would be more helpful would be coming to help us (...) When we're drowning and barely keeping our head above water and the unit is falling apart at the seams, pitching in saying I'll stay at the desk, you go and help out your nurses, go help them get off to break (…) that would be something that would be far more tangible and in the moment be much more helpful. (P5)

Participants believed they should have been given access to more areas to use as break rooms, given social distancing rules. Additionally, it was suggested that offering quick 5-min breaks would have been beneficial to help with things like getting to the bathroom or having a drink of water.

#### Improving communication

Participants noted they struggled to stay updated with the constantly changing policies and found the updates sent by email to be ineffective. Suggestions were made to create a central streamlined communication system. For example, sending updates through the electronic medical record system used throughout the hospital. Other suggestions included having team huddles while on shift to go over any updates or sending them through the hospital's printer system so changes could get posted as soon as they were made. Overall, participants believed there needed to be more transparency and open communication between management and frontline labour and delivery employees.

Transparency was a big one and releasing information, even if you didn't have an answer, saying that you didn't have an answer - being transparent that we acknowledge that this is a concern, and we are going to get you an answer. (P17)

#### Improving retention

Many obstetrical nurses were choosing to change professions or leave the hospital during the COVID-19 pandemic. Suggestions to improve retention included offering more flexibility in scheduling, offering a greater number of job share positions, and making it easier to work only days or only nights if desired. Participants believed that offering workshops or mock training days could have helped retain perinatal nurses by making them feel more confident in their roles and prepared for anomalous situations that occurred in labour and delivery departments during the pandemic. Above all else, participants highlighted that to help with retention, staff appreciation needed to be improved. Often this was linked to simply listening and considering the concerns of frontline obstetrical nurses, acknowledging how difficult these past years have been, and attempting to provide more physical help on the unit to relieve nurse burden. Specific suggestions to help perinatal nurses feel more appreciated during the pandemic included covering the nurses' yearly licensing fees, offering personalized gifts during Nursing Week, and increasing wages or providing incentives when being required to work short.

#### Positive feedback

Participants highlighted a few areas which they believed the hospital handled well in response to the pandemic. Positive comments were made about the hospital limiting the number of visitors permitted on site, which helped reduce virus exposure and ease flow on the unit: "I did enjoy the restricted visitors, not having people in and out all the time and through the hallways made me feel safer and a little bit better" (P18). Closely linked to reduced visitors was the screening put forth at hospital entrances, which participants also acknowledged as a good idea. When asked about positive feedback, it was common for participants to acknowledge that the hospital is restricted to a certain degree by provincial and federal regulations; therefore, it was noted that many things were beyond the hospital's control. Consequently, participants suggested the need for a top-down change, which would include changes to funding and various nursing regulations starting at the provincial level and moving down through the regional health authorities, hospitals, and finally hospital upper management.

#### Summary of results

The results identified four main themes related to the COVID-19 working experiences of obstetrical nurses in an Ontarian tertiary care centre and were grouped based on the JD-R framework (see Fig. 1). Job stressors, which are overwhelming job demands, have a negative impact, both physical and emotional, on frontline obstetrical nurses. This negative impact can be mitigated by job resources as well as personal resources. However, participants did not feel they were adequately supported by upper management during the COVID-19 pandemic. Thus, rather than listing specific job resources, the model displays key improvement areas surrounding job resources, as described by participants.

## Discussion

The findings from this study help to better understand the physical, psychological, social, and organizational aspects that obstetrical nurses struggled with at work during the COVID-19 pandemic. Specifically, the findings show that obstetrical nurses faced challenges with the following job stressors: increased workload, fear of COVID-19 transmission, providing proper patient care to labouring and expecting individuals, and overwhelming physical demands. Participants shared distinct COVID-19 work-related stressors in the field of obstetrics, including heightened COVID-19 exposure due to hospital-wide triage protocols for pregnant individuals, increased workload from the lack of support person(s) for birthing parent, and increased concerns about potential COVID-19 transmission to vulnerable mothers and newborns lacking sufficient immunity. Moreover, having to work short was nearly unavoidable as the unique skillset needed for labour and delivery made it difficult for nurses from other departments to step in as substitutes. It is worth noting that several stressors identified in this study are consistent with the JD-R model. Specifically, increased workload and overwhelming physical demands are well-established job stressors found to coincide with burnout, depersonalization, and absenteeism [30]. Moreover, fear of COVID-19 transmission has been found to be associated with emotional exhaustion when conceptualized as a job demand in the context of the JD-R model [18]. Interestingly, understanding that nurses are worried about their ability to provide proper patient care can help position this phenomenon as a job stressor in the context of the JD-R model and may suggest that nurses who feel they are unable to meet their duty in providing safe, compassionate, and ethical care are at an increased risk of developing psychological problems. In summary, the identified stressors should be recognized as risk factors that contributed to health problems, increased job stress, and decreased job satisfaction among obstetrical nurses during the COVID-19 pandemic.

Participants described the impact, both physical and emotional, of working as a nurse in labour and delivery during the COVID-19 pandemic. Specifically, they described feeling burned-out, undervalued, inadequately supported, a need to make career modifications, and a general concern over the future of healthcare. Although healthcare worker burnout during the COVID-19 pandemic has been documented [31, 32], this study helps to better understand the circumstances surrounding burnout among Ontarian obstetrical nurses working in tertiary care centres. Specifically, findings show participants experienced a decrease in job satisfaction, lack of motivation, lack of sleep, and a decrease in empathy towards patients. Feeling underappreciated, undervalued, and disposable were emotions also frequently mentioned by participants. This finding is consistent with Altman et al.'s [1] study involving 14 perinatal nurses from the United States that found nurses were feeling devalued and expendable during the COVID-19 pandemic. However, the findings in this study indicate that feeling undervalued as a nurse could have been exacerbated if working in the field of labour and delivery given participants noted that they felt their department was often overlooked by upper management. These findings suggest it may be beneficial for hospital upper management to work closely with each department to ensure policy updates are properly disseminated and vaccinations are offered in an order which will help all staff feel safe and protected.

It is crucial to understand that job stressors have a direct effect on burnout, absenteeism, and other negative psychological outcomes. However, such adverse effects can be alleviated through the implementation of appropriate job resources [16]. Yet, participants frequently emphasized that they felt inadequately supported by the hospital over the course of the COVID-19 pandemic, suggesting that the needs of nurses working in the field of obstetrics were commonly overlooked or misunderstood. Moreover, many participants disclosed that working during the COVID-19 pandemic had resulted in them considering a career change or departure from the hospital. This is particularly interesting considering that many participants also frequently highlighted their love for working in labour and delivery, and may suggest that working conditions had simply become unbearable. Together, the findings from this section suggest that the job resources provided to perinatal nurses during the COVID-19 pandemic were not sufficient to alleviate the negative impacts of the job stressors they encountered.

This study found that relying on personal hobbies, support from family, friends and colleagues, career modifications, and their own love for the job were mechanisms participants personally used to help them manage the increased job stress caused by the pandemic. Existing literature has also shown that social support is one of the most effective ways individuals can cope with stress and it can come either from relatives, friends, a spouse, or co-workers [33]. However, it is interesting to note that participants in our study frequently highlighted that support from colleagues was arguably the most beneficial form of social support. They noted this was namely due to the fact that they were able to easily empathize with one another given their shared similar experiences. These findings suggest that the experiences of obstetrical nurses were unique in the sense that outsiders may not have been able to comprehend the extent of their hardships. Additionally, given the nurses' high level of appreciation for their colleagues' support, the findings in our study could point to a need for hospitals to implement peer-support programs. Participants often emphasized their love for being a labour and delivery nurse and described it as a strong motivational factor to continue working through such difficult times. Existing literature shows that healthcare workers with a sense of "higher calling" and commitment to their work are more resilient to burnout [34]. However, to date, there have been no studies which identified a sense of higher calling among labour and delivery nurses during the COVID-19 pandemic as a possible coping mechanism. Given that "love for the job" could be indicative of a sense of "higher calling", further research may be warranted to gather a deeper understanding of this concept. Provided that personal resources have been found to buffer the negative effects of job stressors [19], the findings of this section could suggest that obstetrical nurses who utilized the resources above and held a great love for working in labour and delivery were able to withstand higher levels of job stress throughout the COVID-19 pandemic.

### Recommendations

Based on the study findings, participant recommendations, and the literature, we make recommendations for practice to improve working conditions for obstetrical nurses during major disease outbreaks (see Table 2). The recommendations focus on organizational stress management interventions [20] as they have been proven to be more effective and provide longer-lasting effects [35, 36].

**Table 2:** Recommendations to improve working conditions for obstetrical nurses during major disease outbreaks

Recommendation	Examples
Provide additional psychological support	• Schedule regular debriefing sessions • Offer onsite counselling sessions with mental health specialists • Provide training workshops on how to improve coping skills
Foster peer support groups	• Implement formal peer support groups by creating a designated peer support role for staff members, providing the designated peer supporters with training, and offering a safe space to host the group sessions [37]
Alleviate physical demands	• Provide self-care stations • Ensure proper breaks are taken and vacation time is offered • Hire extra staff to complete non-nursing tasks (i.e., personal support workers or patient care technicians)
Improve communication	• Create a central system to easily update staff • Conduct group huddles while on shift
Allow flexibility in work schedules	• Offer a greater amount of job share positions • Implement shorter shift times for the duration of the outbreak
Provide disease training	• Offer simulation training • Provide up-to-date training on how to properly don and doff personal protective equipment
Increase staff appreciation	• Offer personalized gifts • Offer pay premiums for times of extreme hardship • Ensure department remains properly staffed and staff are provided with proper equipment to safely perform their job
Retain staff	• Create a safe and positive working environment • Form a task force that is focused solely on nurse retention/recruitment [38]

### Limitations

The following limitations need to be considered. Firstly, given that statistical inference is not the purpose of qualitative research, and the recruited participants were from labour and delivery departments within one Ontarian tertiary care centre, the results of this study are not generalizable. However, we believe the high-level themes discovered are transferable and relevant to obstetrical nurses in other Ontario hospital, healthcare settings where nursing shortages were experienced, and within jurisdictions that enacted similar policies to Ontario. For example, the Canadian Nurses Association has also identified increased workload and overwhelming physical demands as contributors to the high rate of burnout seen among nurses during COVID-19 and has underlined the importance of improving working conditions for nurses Canada-wide [39]. Another limitation that must be considered is the fact that interviews conducted at a single point in time may fail to capture feelings, experiences, and perceptions as they evolve over time [40]. Therefore, findings may be specific to how nurses felt at the time of data collection (i.e., during the Omicron wave of the pandemic). Lastly, the study sought to provide an in-depth exploration of COVID-19 job stressors among obstetrical nurses given the impact on one's health and the proper functioning of the healthcare system. However, there may be other factors, such as personal demands, that also had an impact on stress levels during the COVID-19 pandemic that the study did not capture. Future research would benefit from investigating the personal circumstances of obstetrical nurses who worked during COVID-19 to gather an all-encompassing understanding of the factors contributing to an increase in stress among this population.

## Conclusions

This study has contributed to the current body of knowledge on the experiences and stressors faced by healthcare workers during major disease outbreaks. Specifically, the use of qualitative methodology and the JD-R model allowed for a rich exploration into the details and complexities of the working conditions and lived experiences of obstetrical nurses during the COVID-19 pandemic. The study provides an in-depth understanding of COVID-19 job stressors unique to obstetrical nurses who worked in Ontarian tertiary care centres. These stressors include an increase in workload due to the lack of support person(s) for birthing parents, the challenge of having to address labouring individuals conceal COVID-19 symptoms to achieve their preferred birthing experience, and the fear of potentially transmitting COVID-19 to vulnerable mothers and newborns lacking sufficient immunity. Additionally, the study was able to provide insight into the personal resources used by obstetrical nurses to help manage the strain of increased job stress and their needs for various job resources.

The perspectives and experiences shared by participants offer a deeper understanding of the factors contributing to job stress and their needs and desires for support. Thus, the study findings could guide hospital management on the development and implementation of job resources which could help mitigate the risk of psychological distress and improve working conditions for those working in maternal care during future waves of the COVID-19 pandemic and other future major disease outbreaks.

## Data Availability

The datasets generated and/or analysed during the current study are not publicly available due to the sensitive nature of the interview questions but are available from the corresponding author on reasonable request.

## Abbreviation

JD-R model: Job Demands-Resources model
